# Evaluating the impact of a small number of areas on spatial estimation

**DOI:** 10.1186/s12942-020-00233-1

**Published:** 2020-09-25

**Authors:** Aswi Aswi, Susanna Cramb, Earl Duncan, Kerrie Mengersen

**Affiliations:** 1 ARC Centre of Excellence for Mathematical and Statistical Frontiers, Queensland University of Technology, Brisbane, Australia; 2 School of Public Health and Social Work, Institute of Health and Biomedical Innovation, Queensland University of Technology, 60 Musk Ave, Kelvin Grove, QLD 4059 Australia

**Keywords:** Bayesian spatial estimation, Conditional autoregressive (CAR), Few areas

## Abstract

**Background:**

There is an expanding literature on different representations of spatial random effects for different types of spatial correlation structure within the conditional autoregressive class of priors for Bayesian spatial models. However, little is known about the impact of these different priors when the number of areas is small. This paper aimed to investigate this problem both in the context of a case study of spatial analysis of dengue fever and more generally through a simulation study.

**Methods:**

Both the simulation study and the case study considered count data aggregated to a small area level in a region. Five different conditional autoregressive priors for a simple Bayesian Poisson model were considered: independent, Besag-York-Mollié, Leroux, and two variants of a localised clustering model. Data were simulated with eight different sizes of areal grids, ranging from 4 to 2500 areas, and two different levels of both spatial autocorrelation and disease counts. Model goodness-of-fit measures and model estimates were compared. A case study involving dengue fever cases in 14 local areas in Makassar, Indonesia, was also considered.

**Results:**

The simulation study showed that model performance varied under different scenarios. When areas had low autocorrelation and high counts, and the number of areas was at most 25, the BYM, Leroux and localised $G = 2$ models performed similarly and better than the independent and localised $G = 3$ models. However, when the number of areas were at least 100, all models performed differently, and the Leroux model performed the best. Overall, the Leroux model performed the best for every scenario especially when there were at least 16 areas. Based on the case study, the comparative performance of spatial models may also vary for a small number of areas, especially when the data have a relatively large mean and variance over areas. In this case, the localised model with *G* = 3 was a better choice.

**Conclusion:**

Detecting spatial patterns can be difficult when there are very few areas. Understanding the characteristics of the data and the relative influence of alternative conditional autoregressive priors is essential in selecting an appropriate Bayesian spatial model.

## Background

Spatial models are widely used in many fields. A common application is disease mapping [1–6] where typically the region is partitioned into *n* neighbouring small areas with the aim of estimating the overall spatial pattern of disease incidence, survival or risk [1, 7]. Bayesian spatial models are commonly used for this problem, with the model comprising a multiplicative relationship of available covariates and an additional random effects term that describes the residual spatial autocorrelation. These spatial random effects are often represented by a conditional autoregressive (CAR) distribution [7]. A range of CAR priors has been proposed, such as the intrinsic conditional autoregressive (ICAR) model [8], the Besag, York & Mollié (BYM) model [8], the Cressie model [9], the Leroux model [10], locally adaptive [11] and localised models [12].

Several of these models have been compared using simulated data. For example, the BYM model was found to perform well when compared against assorted mixture models, non-parametric smoothing methods and some partition models [1, 13]. However, when the BYM model was compared against both Leroux and Cressie models, the Leroux model was preferred due to its flexibility in representing a range of spatial correlation scenarios [7]. In another study, the BYM, Leroux and locally adaptive models were compared, and the locally adaptive models were found to perform better [11]. In another study which modelled the relative risk of HIV in India, the Poisson log-normal model and various CAR models such as BYM CAR, ICAR, proper CAR [14], Leroux CAR and dissimilarity CAR [15] models were compared [16]. The authors found that the BYM model was the best among all the models considered based on the deviance information criterion (DIC) values.

A common feature in all these comparisons is that there has been a relatively large number of areas in the study region (range: 170 to 271). However, this is not always the case. There is a growing number of studies that employ spatial models over regions with comparatively few areas. This may affect the estimation of the spatial parameters in the above models.

In this paper, we investigate the relative performance of Bayesian spatial models when there are few areas via a simulation study. In keeping with the disease mapping theme, we focus on a Poisson likelihood and consider the BYM, Leroux and localised priors. For reference, the corresponding independent model which ignores spatial autocorrelation is also included. A case study of dengue incidence across the 14 geographic regions of Makassar, Indonesia, is also considered. The overall aim is to evaluate which spatial models are likely to be appropriate for a small number of areas under different scenarios.

## Methods

### Simulated data

A simulation study was designed to examine the impact of a small number of areas on model goodness-of-fit of spatial models using various Bayesian spatial model specifications. Based on the aim of this paper, 50 replicate datasets of disease counts were generated under each of 32 scenarios: eight different numbers of regular areal grids (2 × 2, 3 × 3, 4 × 4, 5 × 5, 10 × 10, 15 × 15, 20 × 20 and 50 × 50), two different degrees of spatial autocorrelation (low, high) and two different levels of counts (low, high). This resulted in a total of 1600 datasets. The size of every areal grid in this simulation is one unit. The total area covered increased as the number of regular areal grids increased.

The following procedure was used to generate the synthetic data. First, an underlying spatial random field (USRF) was generated using a Gaussian decay function with bandwidths 0.1 and 3 for low and high degrees of autocorrelation, respectively. Modified Moran’s I (MMI) was calculated to confirm that the higher bandwidth produced a larger degree of spatial autocorrelation as desired. For numerical stability, the USRF was generated on the log scale, and rescaled to ensure an adequate effect size is achieved. We calculated the SIRs directly from the USRF. SIR values were generated by generating the underlying spatial random field (USRF), as can be seen in step 1 of the R code (Additional file 1). The descriptive summary of counts and SIRs from the simulated data for every scenario are given in Additional file 2.

Second, pseudo observed values $\left( {y_{pseudo} } \right)$ were generated for each area using a gamma distribution. These values were subsequently ordered to resemble a similar spatial pattern to the USRF.

Third, pseudo expected values ($E_{pseudo}$) were computed by using $E_{pseudo} = y_{pseudo} /USRF$.

Fourth, to get observed value ($y$), the pseudo observed values are rescaled to match the desired number of counts per area on average, $y_{av}$, using values 1 and 10 for low and high counts respectively. This was subsequently rounded to an integer value (which may cause small deviations from the total number of desired counts, $y_{av} \times 1$ and $y_{av} \times 10$ for low and high counts, respectively). As a result, the low and high levels of count data had mean values around 1 and 10, respectively.

Lastly, to get the expected value ($E$), the pseudo expected counts were rescaled so that the sum of the observed and expected counts are equal, that is, sum (*y*) = sum(*E*).

We devised this method of simulating spatial data specifically to allow control over the range of the USRF and SIR values (effect size), the average observed counts per area (low, high), and the degree of spatial autocorrelation, while maintaining the necessary constraints like sum(*y*) = sum(*E*), *y* is discrete, and *y/E* reflects the same pattern as the USRF at least to a large degree. No existing method of simulating spatial data that we are aware of allows this level of control while satisfying these conditions.

**Fig. 1:**
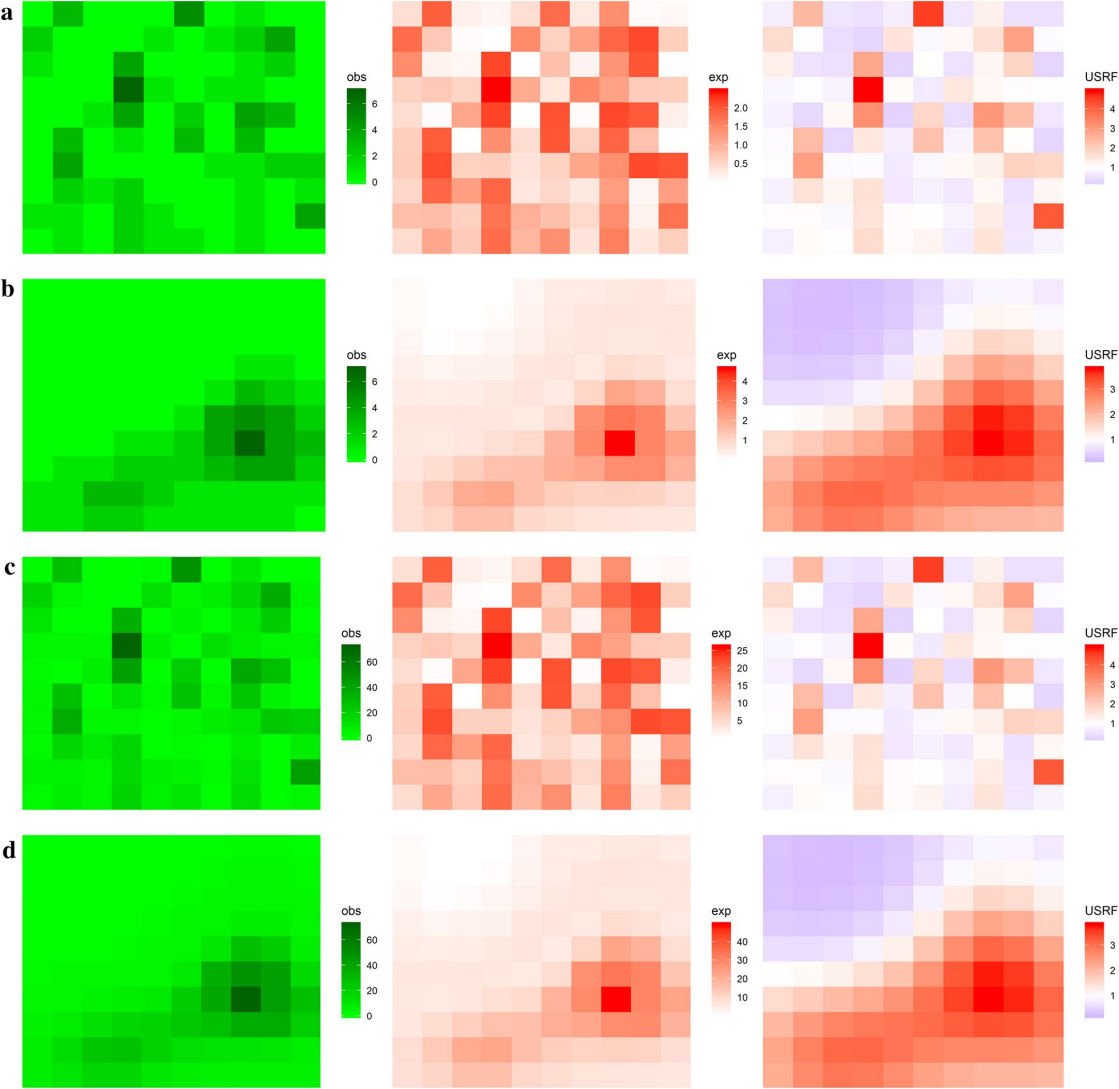
Example results for simulated datasets for 100 areas under different scenarios. **a** low spatial autocorrelation and low counts, **b** high spatial autocorrelation and low counts, **c** low spatial autocorrelation and high counts, and **d** high spatial autocorrelation and high counts. Obs, exp, USRF represented observed values, expected values, and underlying spatial random field, respectively

R code used to generate synthetic data are provided as supplementary information (see Additional file 1). Examples of generated datasets under different scenarios for 10 × 10 areas with low and high spatial autocorrelation and low and high counts are given in Fig. 1. Figure 1a, c, with bandwidth 0.1, exhibit spatial independence (randomness), while Fig. 1b, d, with bandwidth 3, showed that values of similar magnitude were clustered together indicating positive spatial autocorrelation.

### Models

A Bayesian Poisson model was formulated as follows. For the $i^{\text{th}}$ area, *i *=1,…, *I.*

$$y_{i} \sim {\text{Poisson}}\left( {E_{i} \theta_{i} } \right)$$

$${ \log }(\theta_{i} ) = \alpha + \psi_{i}$$

where $y_{i}$ is the observed number of cases; $E_{i}$ is the expected number of cases; and $\theta_{i}$ is the standardised incidence ratio (SIR) which is modelled on the log scale by an overall constant rate, α, and a corresponding residual $\psi_{i}$. These residuals were either modelled as independent and normally distributed (independent model below) or as a combination of independent and spatial random effects, with the latter described by a CAR prior. This prior provides a sparse approximation of the covariance matrix by specifying a local neighbourhood for each area and a corresponding adjacency weight matrix with elements $w_{ij}$ corresponding to the $i^{\text{th}}$ and $j^{\text{th}}$ areas. We follow a common setup for disease mapping, with a first order neighbourhood structure (Queen’s adjacency) and binary spatial weights, such that areas that share a common boundary are considered to be neighbours with $w_{ij} = 1$, and $w_{ij} = 0$ otherwise. Three forms of spatial CAR priors were considered, namely the BYM, Leroux and localised models. All models are described below and were fit using the CARBayes package version 5.0 [15] in R version 3.6.1 [17]. Convergence of each model parameter was checked via the Geweke diagnostic [18], where a *p* value > 0.05 indicated likely convergence, and supplemented by trace and density plots for a subsample of SIRs for each model.

### Independent model

The independent model has no specific term to describe any spatial autocorrelation, so the residual is simply modelled by an unstructured spatial random effect, $v_{i}$ [15]. Under this model,

$$\psi_{i} = v_{i}$$

$$v_{i} \sim {\text{N}}\left( {0,\tau_{v}^{2} } \right).$$

The prior for the variance $\tau_{v}^{2}$ was specified by a vaguely informative inverse Gamma distribution, ${\text{InverseGamma}}\left( {1, 100} \right)$, which is consistent with the implied prior belief that the data do not exhibit spatial autocorrelation.

### BYM model

Possibly the most well-known Bayesian spatial model for areal disease mapping is the BYM model [8]. Under this model,

$$\psi_{i} = u_{i} + v_{i}$$

where $u_{i}$ and $v_{i}$ are the structured and unstructured spatial random effects for area $i$ respectively. As above, a normal distribution was specified for $v_{i}$, i.e., $v_{i} \sim {\text{N}}\left( {0,\tau_{v}^{2} } \right)$. An intrinsic conditional autoregressive (ICAR) prior distribution was specified for $u_{i}$, such that

$$\left( {u_{i} |\varvec{u}_{j} , i \ne j, \tau_{u}^{2} } \right) \sim {\text{N}}\left( {\frac{{\mathop \sum \nolimits_{j} u_{j} w_{ij} }}{{\mathop \sum \nolimits_{j} w_{ij} }}, \frac{{\tau_{u}^{2} }}{{\mathop \sum \nolimits_{j} w_{ij} }}} \right).$$

The variance terms $\tau_{u}^{2}$ and $\tau_{v}^{2}$ were assigned weakly informative priors, namely ${\text{InverseGamma}}\left( {1, 0.01} \right),$which is the default hyperprior specification in CARBayes [15].

### Leroux model

Whereas the BYM CAR model contains two sets of random effects, the Leroux model has only a single spatial random effect, $u_{i}$, that adjusts the strength of the local neighbourhood spatial autocorrelation by a constant $\rho$. The model is thus given by

$$\psi_{i} = u_{i}$$

$$\left( {u_{i} |\varvec{u}_{j} , i \ne j, \tau_{u}^{2} } \right)\sim N\left( {\frac{{\rho \mathop \sum \nolimits_{j} u_{j} w_{ij} }}{{\rho \mathop \sum \nolimits_{j} w_{ij} + 1 - \rho }}, \frac{{\tau_{u}^{2} }}{{\rho \mathop \sum \nolimits_{j} w_{ij} + 1 - \rho }}} \right).$$

with hyperpriors given by $\tau_{u}^{2} \sim {\text{InverseGamma}}\left( {1, 0.01} \right)$ and $\rho \sim {\text{Uniform}}\left( {0, 1} \right)$.

When $\rho = 1$ this prior reduces to the intrinsic CAR model and when $\rho = 0$ it reduces to the independent model [15].

### Localised models

The localised model, proposed by Lee and Saran [19], allows the neighbourhood random effects to markedly differ across the geographic space. This is achieved by partitioning the areas into a maximum of $G$ groups and including a group-specific intercept in the model. An intrinsic CAR prior allows for spatial smoothing across the surface, while the group intercept allows for adjacent areas in different groups to be considered disjoint, enabling distinctly different values. The model is given by

$$\psi_{i} = u_{i} +\lambda_{Z_{i} }$$

$$\left( {u_{i} |\varvec{u}_{j} , i \ne j, \tau_{u}^{2} } \right)\sim {\text{N}}\left( {\frac{{\mathop \sum \nolimits_{j} u_{j} w_{ij} }}{{\mathop \sum \nolimits_{j} w_{ij} }}, \frac{{\tau_{u}^{2} }}{{\mathop \sum \nolimits_{j} w_{ij} }}} \right).$$

where $\tau_{u}^{2} \sim {\text{InverseGamma}}\left( {1, 0.01} \right)$ and $\lambda_{j} \sim {\text{Uniform}}\left( {\lambda_{j - 1} ,\lambda_{j + 1} } \right)$ for $j = 1, 2, \ldots , G$. The group means $\lambda_{1} < \lambda_{2} < \lambda_{3} < \ldots < \lambda_{G}$, where $\lambda_{0} = - \infty$ and $\lambda_{G + 1} = + \infty$. A latent variable $Z_{i}$ determines the allocation of the $i^{\text{th}}$ area to a group,

$$f\left( {Z_{i} } \right) \propto \exp ( - \delta (Z_{i} - G^{*} )^{2} )$$

where the penalty parameter $\delta \sim {\text{Uniform}}\left( {1, 10} \right),$ and $G^{*} = \left( {G + 1} \right)/2$ and $G^{*} = G/2$ if $G$ is odd and even, respectively. The value of $G$ is fixed and the recommendation is for it to be small odd value [19].

### Comparing models

The goodness-of-fit of the models was compared using the Watanabe-Akaike Information Criterion (WAIC) [20]. A smaller value of WAIC indicates a better model fit. The Modified Moran’s I (MMI) [21], which can measure spatial autocorrelation even for small numbers of areas (less than 100), was also calculated using the residuals in order to assess model adequacy, using a confidence level of 0.05. The MMI follows a *t*-distribution with *n*–2 degrees of freedom [21]. A MMI estimate close to zero indicates that the model has appropriately accounted for any spatial structure [2]. The modelled standardised incidence rate (SIR), $\theta_{i}$, was also estimated for each area. The posterior SIR median and 95% credible interval could vary across model scenarios if there is a relatively large contrast counts (observed or expected) between areas.

### Case study

The five models described above were applied to a substantive case study of dengue fever in 2002, 2010, 2011, 2013 and 2015 from Makassar, Indonesia. These years were selected to provide both similarity (in the absolute MMI estimate) and contrast (in the counts) to the simulated scenarios. The annual number of reported dengue cases was obtained for these years in each of the 14 geographic areas of Makassar. The area of Makassar is 175.77 square km with a population of approximately 1.5 million in 2017 (737,146 males and 751,865 females). Across the 14 geographic areas, resident population numbers ranged from 28,696 to 208,436 (median: 99,246) [22]. The data were provided by the City Health Department of Makassar, South Sulawesi Province.

## Results

### Simulated data

The simulated data for low counts had a mean value around 1, with maximum values ranging from 2 to 14, median values ranging from 0 to 1.5, and variance ranging from 0.92 to 4.12. The simulated data for high counts had a mean value around 10, with maximum values ranging from 16 to 140, median values ranging from 3 to 11, and variance ranging from 44.67 to 387.13. Area-level analyses of dengue fever would often have both high and low counts present and, particularly for high counts and as the numbers of areas increase, this diversity of counts across areas is present in our simulated data (see Additional file 2).

**Fig. 2:**
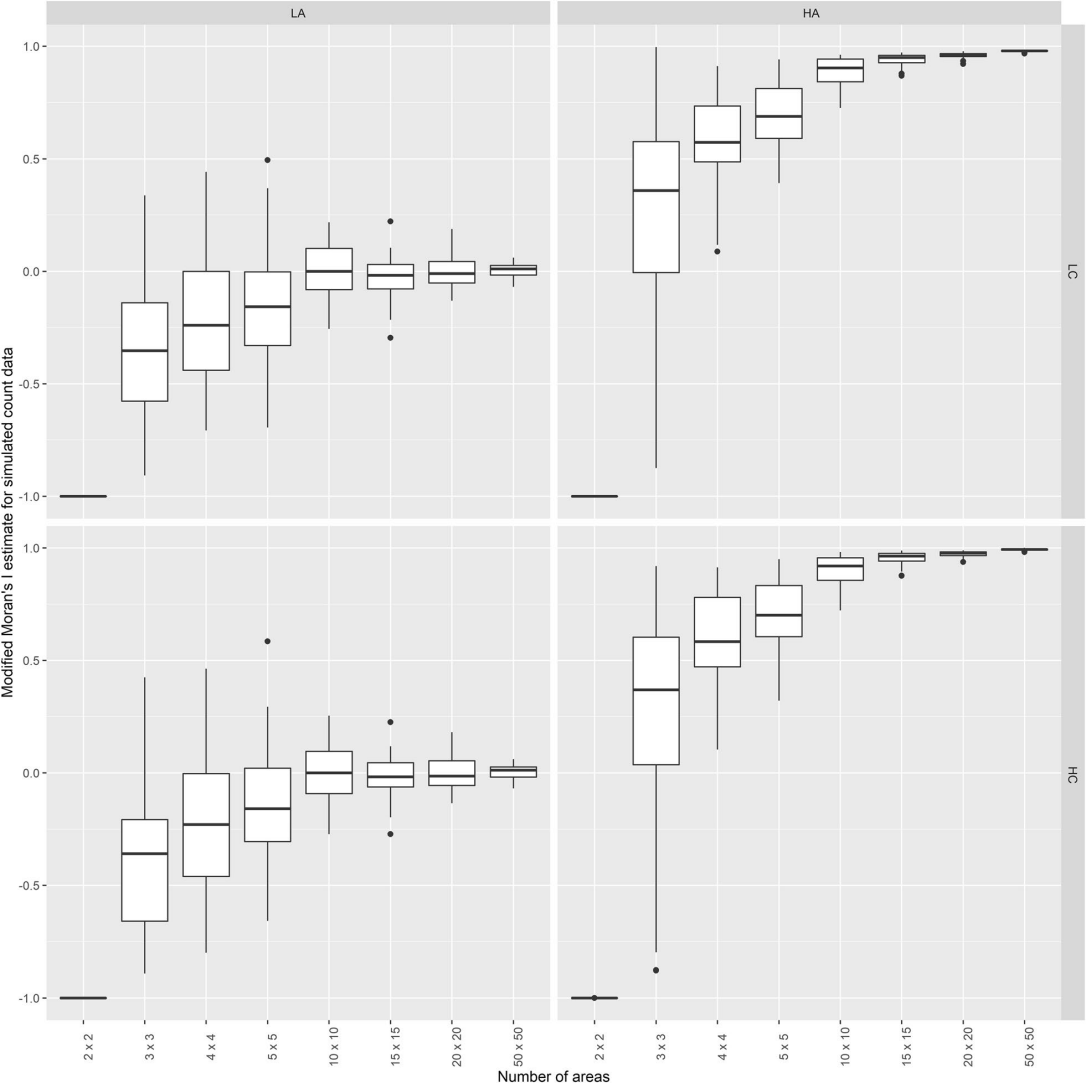
Modified Moran’s I estimates for simulated count data. *LA LC* low spatial autocorrelation and low counts, *HA LC* high spatial autocorrelation and low counts, *LA HC* low spatial autocorrelation and high counts, *HA HC* high spatial autocorrelation and high counts

Box-plots of MMI estimates for the 50 replicates of data over the 32 different scenarios are shown in Fig. 2. Figure 2 indicates that MMI, a measure of spatial autocorrelation, performs poorly when there are very few areas (2 × 2), but tends to capture especially high spatial autocorrelation even when the number of areas is relatively small (4 × 4, and 5 × 5 areas). MMI estimates for low autocorrelation are close to zero, while the MMI estimate for high autocorrelation are close to one.

**Fig. 3:**
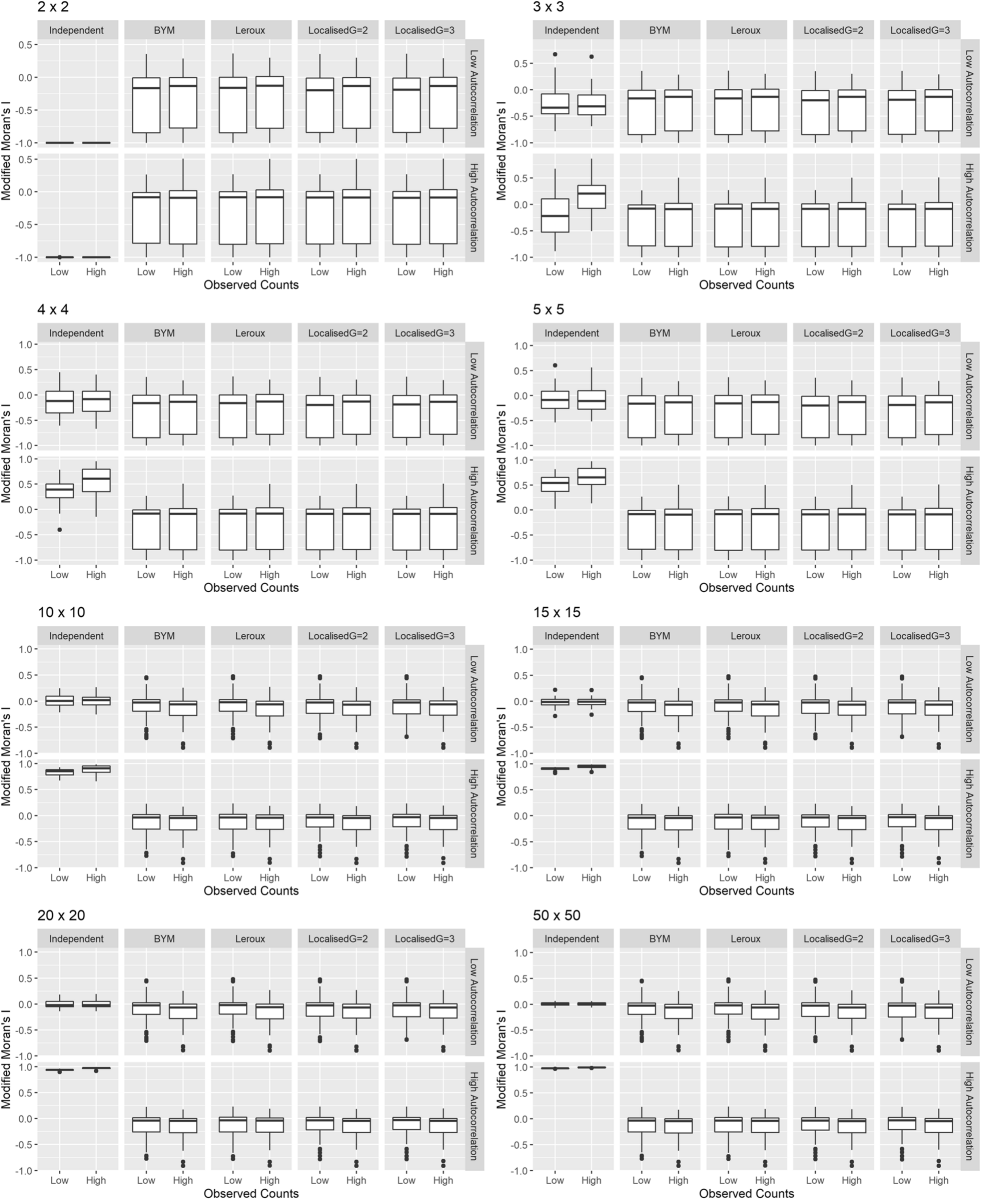
Modified Moran’s I estimates for residuals

The residuals for the five models were also tested for spatial autocorrelation using MMI statistics. The results are depicted in Fig. 3. It can be seen that when there is strong spatial structure in the data, the test correctly identifies spatial autocorrelation in the residuals of the independent model but not in the residuals of the spatial models. However, this differential is not as clear when the autocorrelation is low and the number of areas is small.

The results of the WAIC values and corresponding 95% credible intervals for the five models are shown in Table 1. Boxplots of the WAIC values are presented as supplementary information (see Additional file 3).

**Table 1:** Mean WAIC values and 95% credible intervals for every model

Areas	Levels	WAIC Mean (95% CI)				
		Independent	BYM	Leroux	Localised G = 2	Localised G = 3
2 × 2	LA LC	8.7 (4.8, 11.8)	*11.3 (6.7, 16.2)*	*11.4 (6.6, 16.3)*	*11.4 (6.6, 16.4)*	*11.1 (6.5, 16.1)*
	HA LC	49.6 (5.0, 206.3)	*12.1 (7.2, 18.0)*	*12.1 (7.2, 17.9)*	*12.1 (7.1, 17.6)*	34.9 (7.1, 180.2)
	LA HC	153.1 (53.7, 358.4)	*79.7 (38.4, 124.3)*	*80.0 (37.7, 122.9)*	*83.5 (36.6, 144.4)*	163.7 (49.2, 506.6)
	HA HC	524.8 (64.7, 1915.3)	*91.6 (41.4, 151.6)*	*91.4 (41.3, 149.3)*	95.2 (41.0, 163.1)	360.2 (54.6, 2170.8)
3 × 3	LA LC	*18.6 (13.4, 23.5)*	*19.6 (14.7, 26.7)*	*19.7 (14.4, 26.8)*	*19.6 (14.5, 26.9)*	*19.6 (14.4, 26.6)*
	HA LC	530.4 (30.6, 446.7)	*19.7 (15.2, 24.9)*	*19.5 (15.1, 24.8)*	*19.5 (14.9, 25.0)*	35.1 (17.4, 81.9)
	LA HC	253.5 (143.9, 420.3)	*112.3 (65.9, 185.6)*	*112.2 (62.0, 187.8)*	*112.5 (62.4, 188.9)*	160.0 (78.1, 364.4)
	HA HC	5397.6 (302.7, 5134.2)	*111.1 (73.7, 166.6)*	*108.0 (68.9, 163.2)*	*108.7 (67.3, 163.3)*	281.0 (98.2, 687.9)
4 x 4	LA LC	*33.5 (24.1, 40.9)*	*32.6 (24.1, 40.9)*	*32.6 (23.9, 40.7)*	*32.6 (24.0, 40.9)*	*32.6 (23.9, 40.9)*
	HA LC	177.3 (73.8, 563.5)	*33.6 (25.5, 43.6)*	*33.3 (25.2, 43.2)*	*36.1 (25.4, 83.3)*	44.5 (28.0, 76.0)
	LA HC	400.2 (244.4, 632.0)	*158.8 (95.5, 240.0)*	*156.4 (93.9, 235.1)*	*158.0 (95.1, 237.1)*	193.5 (103.9, 310.0)
	HA HC	1820.7 (645.1, 6367.0)	168.5 (105.1, 272.7)	162.9 (101.2, 263.1)	195.3 (101.6, 748.3)	280.1 (125.0, 568.1)
5 x 5	LA LC	50.7 (39.4, 60.6)	*46.4 (36.8, 60.4)*	*46.4 (37.0, 60.6)*	*46.4 (37.0, 60.6)*	*46.4 (36.9, 60.5)*
	HA LC	270.7 (111.8, 443.2)	*48.6 (35.7, 62.9)*	*48.2 (35.5, 62.6)*	*48.9 (35.4, 68.4)*	219.9 (39.8, 115.9)
	LA HC	530.1 (377.7, 717.7)	*205.5 (149.1, 308.3)*	*202.9 (150.0, 300.7)*	*203.4 (149.6, 298.9)*	225.8 (158.3, 329.2)
	HA HC	2795.1 (1116.3, 4045.0)	226.6 (135.9, 351.9)	217.6 (132.6, 333.1)	227.0 (130.5, 393.5)	2169.8 (156.8, 1011.9)
10 x 10	LA LC	194.4 (175.2, 214.8)	*166.4 (150.2, 178.9)*	*166.1 (150.3, 178.4)*	*166.3 (150.3, 178.4)*	*166.2 (150.2, 182.4)*
	HA LC	479.2 (408.2, 563.8)	*186.6 (157.5, 216.7)*	*185 (155.5, 215.6)*	190.3 (158.7, 269)	449.5 (158.7, 222.2)
	LA HC	1460.1 (1349.4, 1557.7)	612.9 (502.5, 723.9)	592.9 (489.7, 713.5)	602.5 (486.8, 741.3)	610.6 (493.2, 724.1)
	HA HC	4326.9 (3530.2, 5334.6)	817.9 (537.3, 1210.9)	771.3 (516.3, 1133.3)	832.8 (520.5, 1753.0)	3548.4 (562.6, 1204.5)
15 x 15	LA LC	426.0 (398.2, 448.8)	*367.4 (346.3, 392.2)*	*366.7 (345.7, 391.4)*	*367.2 (346.0, 392.6)*	*367.0 (345.6, 391.6)*
	HA LC	817.0 (727.8, 905.9)	*404.9 (354.7, 449.2)*	*402.2 (351.9, 445.7)*	410.2 (354.3, 446.2)	411.9 (359.8, 482.4)
	LA HC	2783.0 (2668.2, 2923.1)	1259.3 (1098.0, 1469.6)	1210.6 (1066.7, 1411.1)	1230.4 (1059.5, 1465.1)	1233.5 (1078.1, 1460.4)
	HA HC	6770.8 (5681.6, 7975.3)	1621.2 (1133.3, 2217.3)	1529.4 (1071.5, 2059.6)	1629.4 (1099.6, 2133.9)	1637.1 (1145.9, 2353.9)
20 x 20	LA LC	737.6 (704.9, 771.7)	*642.1 (611.7, 671.2)*	*640.9 (610.8, 670.0)*	*641.7 (610.9, 670.0)*	*641.5 (610.6, 670.1)*
	HA LC	1227.4 (1139.6, 1304.1)	*697.8 (626.7, 778.1)*	*694.2 (625.1, 774.8)*	713.6 (625.1, 850.5)	707.9 (627.1, 787.4)
	LA HC	4374.6 (4240.8, 4543.4)	2106.5 (1836.8, 2351.5)	2029.9 (1784.1, 2246.3)	2062.7 (1786.8, 2359.2)	2063.2 (1802.1, 2325.0)
	HA HC	9425.4 (8401.6, 10431.7)	2613.9 (2020.9, 3419.3)	2483.1 (1972.5, 3207.7)	2713 (1954.2, 4157.8)	2632.9 (1992.7, 3451.8)
50 x 50	LA LC	4222.5 (4153.0, 4305.9)	3880.0 (3757.4, 3981.6)	*3875.3 (3753.6, 3979.0)*	*3877.5 (3757.8, 3979.3)*	*3876.8 (3752.9, 3977.8)*
	HA LC	5315.6 (5107.9, 5514.9)	4064.8 (3901.2, 4234.0)	4054.3 (3886.6, 4217.4)	4060.1 (3890.3, 4217.1)	4076.6 (3896.9, 4360.9)
	LA HC	18863.8 (18370.4, 19290.3)	11508.6 (10749.7, 12251.0)	11224.8 (10577.2, 11922.3)	11415.1 (10696.4, 12376.5)	11314.8 (10541.2, 12212.4)
	HA HC	30471.4 (27777.6, 32943.7)	13151.4 (11601.2, 14633.0)	12731.1 (11244.9, 13969.9)	12918 (11312.7, 14738.7)	12977.2 (11292.3, 15994.3)

*WAIC* Watanabe-Akaike Information Criterion, *LA LC* low autocorrelation and low counts, *LA HC* low autocorrelation and high counts, *HA LC* high autocorrelation and low counts, *HA HC* high autocorrelation and high counts

Underline represents the lowest mean WAIC out of the 5 models

Italics per row indicates that models perform similarly

Overall, based on the mean WAIC (Table 1), when areas had low autocorrelation and high counts, models performed differently for all number of areas. Interestingly, when the number of areas is at most 5 × 5, the BYM, Leroux and localised $G = 2$ models often performed similarly, but noticeably better than the independent and localised $G = 3$ models. However, when the number of areas were at least 10 × 10, all models performed differently, with the Leroux model having the best goodness-of-fit according to WAIC.

The Leroux model had the best fit, and the independent model had the worst model fit for every scenario especially when the number of areas at least 16 areas based on the WAIC.

When the simulated data had low autocorrelation and low counts, the models performed differently for at least 5 × 5 areas. In this scenario, the independent model had a worse model fit than the remaining four models, which had a similar model fit to each other.

### SIR estimates

**Fig. 4:**
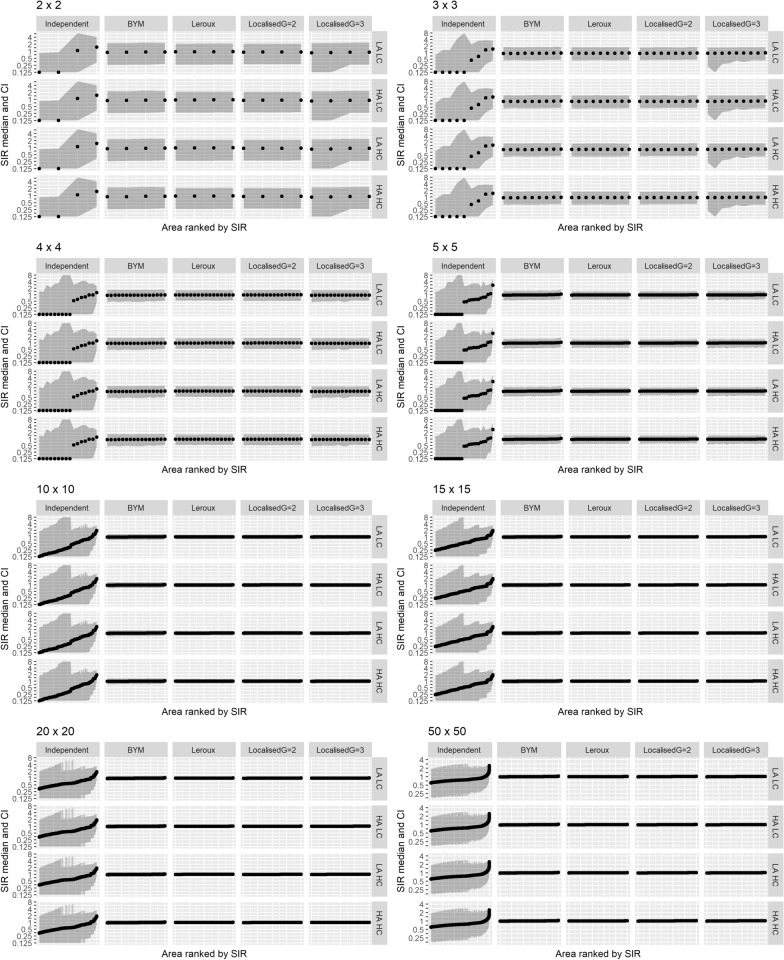
The posterior medians and 95% credible intervals of SIR based on the simulated data. *LA LC* low autocorrelation, low counts; *LA HC* low autocorrelation, high counts. *HA LC* high autocorrelation, low counts; *HA HC* high autocorrelation, high counts

The posterior median and 95% credible intervals of the SIR for each model for every scenario are given in Fig. 4.

Generally, the independent model results differed from the other four models. As expected, this model tended to produce more extreme SIR estimates (further from one) and wider credible intervals under every scenario. This is partly due to the relatively vague prior, which reduces the amount of shrinkage in the spatial random effect, but may be also influenced by the shrinkage towards the global mean of zero rather than the local neighbourhood mean.

### Case study: dengue fever incidence in Makassar, Indonesia

Dengue fever diagnoses fluctuate between years and areas in Makassar, Indonesia. The descriptive analysis of dengue cases in 2002, 2010, 2011, 2013, and 2015 are given in Table 2.

**Table 2:** Descriptive analysis of dengue cases in 2002, 2010, 2011, 2013, and 2015

Year	Dengue cases	Min	1st Qu	Median	Mean	3rd Qu	Max	Var	MMI estimate
2002	1467	14	44.00	86.00	104.80	98.00	419	10622.80	−0.36
2010	185	1	6.25	8.50	13.21	16.75	45	147.26	−0.36
2011	85	1	2.00	3.00	6.07	9.25	16	29.46	−0.19
2013	265	4	9.00	13.50	18.93	27.75	52	210.22	0.19
2015	142	2	4.75	8.00	10.14	14.00	26	50.59	−0.20

From Table 2, it is apparent that there is substantial variation both within and between years, with the mean and variance of dengue cases in 2002 much larger than the other years. In 2002, a total of 1467 cases were diagnosed with a median of 86 cases per area. In this year, one area (Rappocini district) has much higher number of dengue cases than the remaining 13 areas. However, in 2010 only 185 cases were diagnosed, with a median of 8.5 cases per area. Each of the years examined had low autocorrelation based on the MMI estimates and high counts.

To obtain the disease risk estimates, raw standardised incidence ratio (SIR) for each area can be used. Raw SIR is the ratio of the observed number of dengue cases (*y*_*i*_) to the expected number of dengue cases in each area (*E*_*i*_) as follows:

$$SIR_{i} = \frac{{y_{i} }}{{E_{i} }}$$

**Fig. 5:**
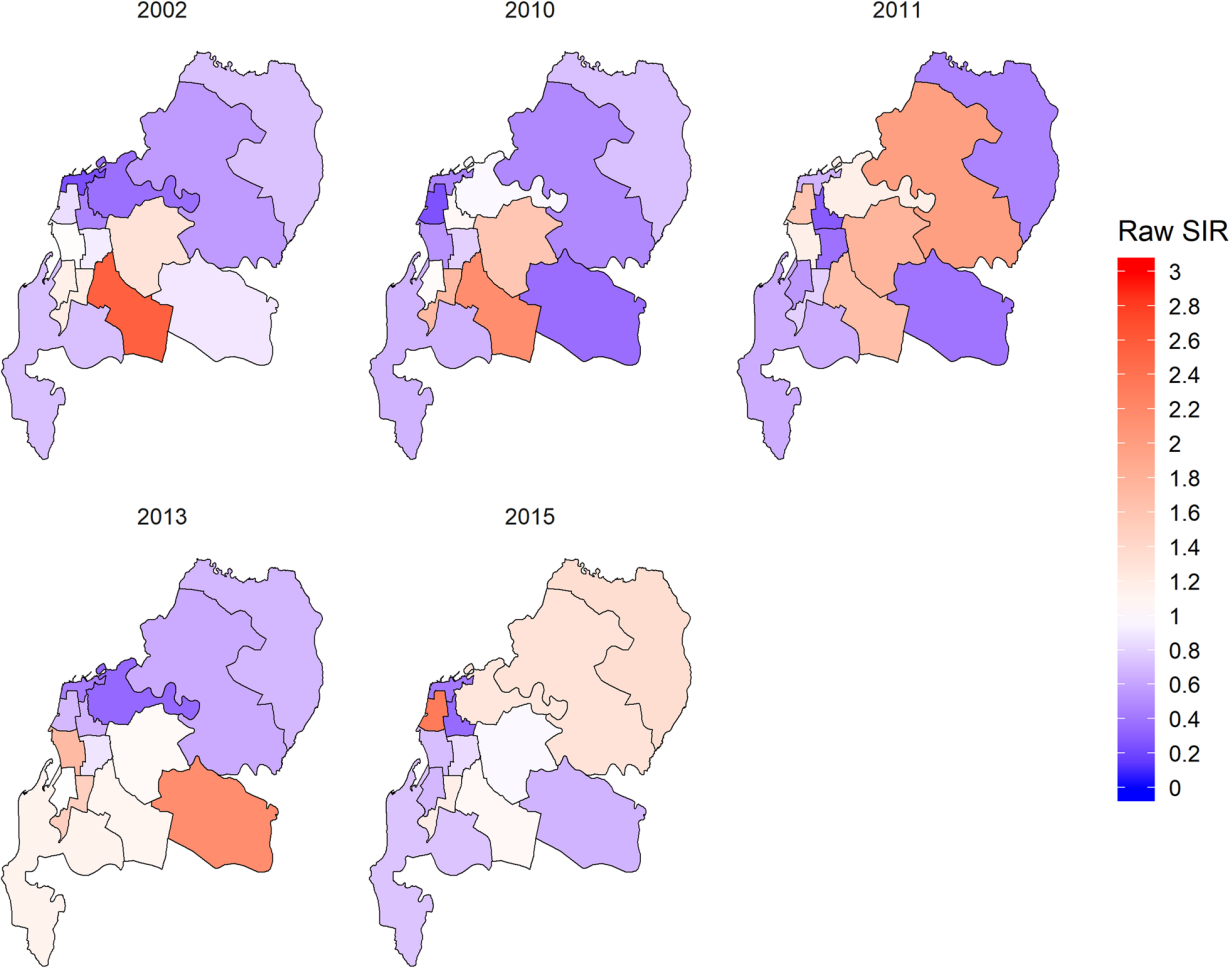
Raw SIR maps for five time periods

Expected number of cases can be calculated as follows:

$$E_{i} = \frac{{\mathop \sum \nolimits_{i} y_{i} }}{{\mathop \sum \nolimits_{i} n_{i} }}n_{i}$$

where $n_{i}$ denoting the number of population at area i. The raw SIR maps for years 2002, 2010, 2011, 2013 and 2015 are given in Fig. 5.

The WAIC were used to compare the goodness-of-fit of the models. The WAIC values of dengue cases for five models are shown in Table 3.

**Table 3:** WAIC of dengue cases for five models

	Model				
	Independent	BYM	Leroux	Localised *G* = 2	Localised *G* = 3
2002	115.14	*114.92*	116.19	175.08	124.54
2010	90.55	84.12	84.98	*79.68*	81.79
2011	78.05	*72.49*	76.04	76.07	75.47
2013	92.17	92.89	91.41	97.23	*91.36*
2015	84.56	73.32	73.59	73.29	73.39

Italics represents the lowest WAIC out of the 5 models

Even though the MMI estimates were the same for dengue cases in 2002 and 2010 (Table 2), the model goodness-of-fit was substantively different (Table 3). Dengue cases in 2002 had a mean (104.80) and variance (10622.80) much higher than the mean (13.21) and variance (147.26) in 2010.

In 2002, Independent, BYM and Leroux model performed similarly, but differed from localised *G* = 2 and localised G = 3 models in terms of WAIC. In this year, the localised model with $G = 3$ performed better than the localised model with $G = 2$. The estimate of $\rho$ for the Leroux model in 2002 was 0.08, indicating that there was very little spatial smoothing.

In 2010, BYM and Leroux models performed similarly but differed from localised and independent models. In this year, the localised model with *G* = 2 performed the best followed by the localised model with *G* = 3.

The localised model with $G = 3$ in 2002 and 2013 consisted of three groups while under *G* = 2 only consisted of two groups. Despite this, modelled SIR estimates were relatively similar between areas under both models (Additional file 4). In both years and models, one area (SIR > 2.0) formed its own group. In 2002 it was Rappocini district, and in 2013 it was Manggala.

In 2010, 2011 and 2015, both localised *G* = 2 and localised G = 3 models comprised the same areas in each group (2, 1 and 1 groups, respectively).

It seems that the number of dengue cases, the variance, and any outliers influence the number of groups as seen in 2002. As mentioned earlier in the case study section, the outlier (Rappocini district in 2002) was a valid observation so was not removed in the analysis.

Dengue data in 2011 and 2015 also had similar MMI estimates (-0.2), but the model goodness-of-fit was substantively different (Table 3). In 2011, the BYM model had the lowest WAIC out of the models considered. The Leroux, localised $G = 2$ and localised $G = 3$ models had a similar model fit based on WAIC, but the independent model gave the highest WAIC out of the models considered. Interestingly, the dengue data in 2015 had similar descriptive statistics with the simulated data for high counts. The observed mean of the dengue cases in this year was 10.14, and the mean simulated data is 10. The BYM, Leroux, localised $G = 2$ and localised $G = 3$ models were similar model fit based on WAIC and provided a better fit than the independent model, which is similar to the results of the simulation study. The estimate of $\rho$ for the Leroux model in 2011 and 2015 was 0.35 and 0.39 respectively, indicating that there was very little spatial smoothing. Both the localised with $G = 2$ and $G = 3$ models in 2011 and 2015, which allows for up to two and three groups of areas respectively, consisted only one group. The SIR of each areas in 2011 and 2015 are relatively similar, that is, around 1 (see Additional file 4).

Furthermore, in 2013, the localised with $G = 3$ model had the lowest WAIC. The estimate of $\rho$ for the Leroux model in 2013 was 0.13, indicating that there was very little spatial smoothing. The localised with $G = 2$ and $G =$ 3 models in 2013 consisted of two groups and three groups, respectively (see Additional file 4).

## Discussion

Five different Bayesian spatial models have been investigated with respect to their ability to provide a good model fit and accommodate spatial autocorrelation in the context of a small number of areas. Our study showed that spatial models may fit the data better even when there is low autocorrelation and low counts, especially when there are at least 25 areas. All of our spatial models allowed for some unstructured variation in the results, and possibly this flexibility is what is driving our results. The estimates of $\rho$ for the Leroux model when using simulated data that had low spatial autocorrelation and high counts ranged from 0.04 to 0.27, closer to 0, indicating some, but weak, spatial autocorrelation. Furthermore, the estimates of $\rho$ for the Leroux model when using simulated data that had low spatial autocorrelation and low counts ranged from 0.19 to 0.50.

Overall, our simulation study showed that the Leroux model performed well for all models under every simulated scenario, especially when there are at least 16 areas. This result is in agreement with another study (with 271 areas) that reported the Leroux model was preferred when compared against BYM and Cressie models [7].

Our case study results largely agree with the simulation study findings, especially for the dengue data in 2015. All Bayesian spatial models (BYM, Leroux and localised models) also performed better (lower WAIC) than the independent model once the number of areas was at least 25. This may be due in part to the localised models often forming just the one group in both simulated and case study data when there were low counts, and 2 groups when counts were high (Additional file 5).

The localised models are a better choice when the counts data and the variation in the counts between areas are high as seen in the 2013 dengue data. Both localised *G* = 2 and *G* = 3 gave very similar SIR values, even when the number of groups differed. However, there is a risk that using an even G value may result in different intercepts even when a smooth risk surface is ideal, so if only one localised model is used, it should be a small odd value [19]. The localised model’s specialty is to allow for large variation between adjacent areas. When this is unnecessary, the extra complexity in this model becomes superfluous, making it a poor choice. One drawback of the localised models is the inherent uncertainty in the forming of groups. Running the same model, under the same specifications and data, can result in slight discrepancies in group assignment. To ensure reproducibility of results, models should be run multiple times, e.g. 5, and the most common grouping used.

Our study also clearly demonstrated the difficulty in detecting spatial patterns when the number of areas is very few (4, or even 9 areas). Even the high autocorrelation and high count data was difficult to distinguish from a random pattern.

Other studies have used 10 sub-districts [23, 24], 30 sub-districts [25], and 38 districts [26] for modelling dengue fever in Indonesia. Most of these papers used ICAR for the spatial structured random effect component. Our findings could be beneficial as a guideline in choosing spatial models in modelling disease in general under different scenarios.

The SIR results were largely similar between the different spatial models, although when there were fewer areas, sometimes differences in estimate precision (as evidenced by the credible intervals) were observed. These differences became vanishingly small as the number of areas increased. This differed from the markedly different results observed for the independent model. This may be influenced in part by the spatial models having the same hyperpriors on their spatially correlated term. Bayesian spatial models are known to be sensitive to the choice of hyperprior, and limitations of software necessitated using the InverseGamma distributions. Ideally, trying different distributions (such as a Uniform prior on the standard deviation [27]), as well as considering different hyperparameters within each distribution, is recommended.

A limitation of this study is that the simulated data may have been influenced by the neighbourhood structure. In terms of the choice of spatial weights, the first-order adjacency specification seems appropriate and has been shown to be a good universal choice for implementing spatial smoothing in general [28]. Additionally, real boundaries generally do not exhibit a grid-style structure, but instead define areas that vary in size, shape and number of neighbours. However, grids have been used in conjunction with real data [29, 30] and the choice of the specification of the spatial weights matrix, being first-order adjacency, is likely to minimise whatever impact this may have. Our approach differentiates between low and high counts. This could be modified to differentiating between low and high SIR values, which of course may be different if the underlying population sizes vary. Our simulation study results are limited by not having a relatively large contras in the magnitude and variation on counts over areas as seen in the case study in certain years. Future work could consider the relatively large variation in the counts between areas. Another limitation of this simulated data is that only means of 1 and 10 were considered as low and high counts, respectively. Often regions are divided into fine geographical resolution, resulting in relatively small average counts, however, if there are average counts markedly greater than 10, this may affect the results.

## Conclusion

In conclusion, the results of this study show that using Bayesian spatial models that allow for both spatial and unstructured correlation can often provide solid and stable results, despite the data characteristics. Nonetheless, detecting spatial patterns will be difficult when there are very few areas. Important data characteristics to consider include the magnitude of the response variable mean and variance, as well as the presence of spatial autocorrelation. Understanding the characteristics of the data and the relative performance of contended models is essential in choosing an appropriate analytic approach.

Care is needed in choosing a spatial model when the number of areas is at least 100, especially when the overall disease rate is high, as models can perform differently.

## Supplementary information

**Additional file 1:** Details and R code for generating the synthetic data.

**Additional file 2:** The descriptive summary of counts and SIRs from the simulated data for every scenario

**Additional file 3:** Box plots of WAIC for different numbers of areas.

**Additional file 4:** The localised structures for localised model with G = 2 and G = 3 in 2002 and 2010; 2011, 2013 and 2015.

**Additional file 5:** The number of groups forming under every scenario.

## Data Availability

All data analysed in the paper except the associated R code, the descriptive analysis of the simulated data, box plots of WAIC, the localised structures for localised model with *G* = 2 and *G* = 3, and the number of groups forming under every scenario are provided as additional files.

## Abbreviations

CAR: Conditional autoregressive
DIC: Deviance information criterion
USRF: Underlying spatial random field
WAIC: Watanabe-Akaike information Criterion
SIR: Standardised incidence ratio
MMI: Modified Moran’s I
