# Supplementary material for: Evaluating the impact of a small number of areas on spatial estimation
Source: Int J Health Geogr. 2020 Sep 25;19:39. doi: 10.1186/s12942-020-00233-1 (PMC7519538; doi:10.1186/s12942-020-00233-1)
Supplement: Supplementary file 1 — Additional file 1: Details and R code for generating the synthetic data. [file 12942_2020_233_MOESM1_ESM.docx]

Additional file

**Additional file 1. Details and R code for generating the synthetic data.**

| # Load packages  library(spdep)  # Decay functions  Gaus.decay <- function(d, bandwith){  exp(-0.5 * (d/bandwith)^2)  }  # Example-specific parameters  N <- 100 # a 10 x 10 regular grid of areas  bandwidth <- 3 # Bandwidth of decay function  r <- 3 # range of log-USRF to control effect size  #(we suggest a value of 3)  y.av <- 1 # Average observed counts per area for low counts  # For this example, d is defined as the distances between  # centroids in the 10 x 10 grid of areas  # 1) **Generate log-underlying spatial random field (USRF)**  log.USRF <- Gaus.decay(d, bandwidth) # Spatial RF with specified autocorrelation  log.USRF <- log.USRF %*% rnorm(N, 0, 10) # Convert N x N SRF to  N-length vector  r.old <- max(log.USRF) - min(log.USRF)  log.USRF <- log.USRF / r.old * r # Rescale so the range equals r  # Although this doesn't need to be centred around zero, it shouldn't be too far from zero  if(max(log.USRF) > r*2/3){  log.USRF <- log.USRF - max(log.USRF) + r/3  }  if(min(log.USRF) < -r*2/3){  log.USRF <- log.USRF - min(log.USRF) - r/3  }  **USRF <- exp(log.USRF)**  # **2) Generate pseudo observed values, y.pseudo**  y.pseudo <- rgamma(N, 0.5, 0.05)  # Reorder y.pseudo to match order of USRF  ord.U <- order(USRF)  ord.y <- order(y.pseudo)  y.pseudo <- y.pseudo[ord.y][order(ord.U)]  **# 3) Compute pseudo expected values, E.pseudo**  E.pseudo <- y.pseudo / USRF  # **4) Rescale y.pseudo so it matches desired level of counts**  y.pseudo <- y.pseudo / sum(y.pseudo) * y.av * N  # 5 **To get observed value y**, round y.pseudo so it is discrete  y <- round(y.pseudo)  # 6 To get Expected value (E),rescale E.pseudo so it satisfies the constraint sum(y) == sum(E)  E <- E.pseudo / sum(E.pseudo) * sum(y) |
| --- |

Example R code simulating the data. This example assumes a 10 x 10 regular grid of areas, where the autocorrelation of the USRF is large (bandwidth = 3), and the average number of cases is low ($y_{av}=1$).
