# Supplementary material for: Evaluating the impact of a small number of areas on spatial estimation
Source: Int J Health Geogr. 2020 Sep 25;19:39. doi: 10.1186/s12942-020-00233-1 (PMC7519538; doi:10.1186/s12942-020-00233-1)
Supplement: Supplementary file 2 — Additional file 2: The descriptive summary of counts and SIRs from the simulated data for every scenario [file 12942_2020_233_MOESM2_ESM.docx]

**Additional file 2. The descriptive summary of counts and SIRs from the simulated data for every scenario**

| Areas | Levels |  | Min | 1^st^ Qu. | Median | Mean | 3^rd^ Qu. | Max | Var |
| --- | --- | --- | --- | --- | --- | --- | --- | --- | --- |
| 4 | LA LC | Observed | 0.00 | 0.75 | 1.50 | 1.25 | 2.00 | 2.00 | 0.92 |
|  |  | Raw SIR | 0.00 | 0.53 | 0.85 | 1.64 | 1.97 | 4.88 | 4.84 |
|  | HA LC | Observed | 0.00 | 0.75 | 1.50 | 1.25 | 2.00 | 2.00 | 0.92 |
|  |  | Raw SIR | 0.00 | 0.59 | 0.96 | 1.45 | 1.82 | 3.90 | 2.87 |
|  | LA HC | Observed | 2.00 | 5.75 | 11.00 | 10.00 | 15.25 | 16.00 | 44.67 |
|  |  | Raw SIR | 0.22 | 0.51 | 0.77 | 1.66 | 1.92 | 4.88 | 4.70 |
|  | HA HC | Observed | 2.00 | 5.75 | 11.00 | 10.00 | 15.25 | 16.00 | 44.67 |
|  |  | Raw SIR | 0.17 | 0.56 | 0.87 | 1.45 | 1.76 | 3.90 | 2.78 |
| 9 | LA LC | Observed | 0.00 | 0.00 | 0.00 | 1.00 | 1.00 | 6.00 | 3.75 |
|  |  | Raw SIR | 0.00 | 0.00 | 0.00 | 0.78 | 1.37 | 2.44 | 1.02 |
|  | HA LC | Observed | 0.00 | 0.00 | 0.00 | 1.00 | 1.00 | 6.00 | 3.75 |
|  |  | Raw SIR | 0.00 | 0.00 | 0.00 | 0.75 | 1.64 | 1.80 | 0.80 |
|  | LA HC | Observed | 1.00 | 2.00 | 4.00 | 10.00 | 6.00 | 58.00 | 329.25 |
|  |  | Raw SIR | 0.14 | 0.17 | 0.60 | 0.79 | 0.82 | 2.36 | 0.59 |
|  | HA HC | Observed | 1.00 | 2.00 | 4.00 | 10.00 | 6.00 | 58.00 | 329.25 |
|  |  | Raw SIR | 0.10 | 0.26 | 0.78 | 0.76 | 0.98 | 1.74 | 0.32 |
| 16 | LA LC | Observed | 0.00 | 0.00 | 0.00 | 0.88 | 0.25 | 6.00 | 4.12 |
|  |  | Raw SIR | 0.00 | 0.00 | 0.00 | 0.43 | 0.38 | 1.93 | 0.60 |
|  | HA LC | Observed | 0.00 | 0.00 | 0.00 | 0.88 | 0.25 | 6.00 | 4.12 |
|  |  | Raw SIR | 0.00 | 0.00 | 0.00 | 0.50 | 0.46 | 2.21 | 0.82 |
|  | LA HC | Observed | 0.00 | 1.75 | 3.00 | 10.06 | 5.00 | 64.00 | 387.13 |
|  |  | Raw SIR | 0.00 | 0.29 | 0.46 | 0.57 | 0.54 | 1.79 | 0.26 |
|  | HA HC | Observed | 0.00 | 1.75 | 3.00 | 10.06 | 5.00 | 64.00 | 387.13 |
|  |  | Raw SIR | 0.00 | 0.20 | 0.42 | 0.63 | 0.85 | 1.75 | 0.34 |
| 25 | LA LC | Observed | 0.00 | 0.00 | 0.00 | 0.96 | 1.00 | 7.00 | 3.12 |
|  |  | Raw SIR | 0.00 | 0.00 | 0.00 | 0.55 | 0.92 | 4.17 | 0.80 |
|  | HA LC | Observed | 0.00 | 0.00 | 0.00 | 0.96 | 1.00 | 7.00 | 3.12 |
|  |  | Raw SIR | 0.00 | 0.00 | 0.00 | 0.57 | 1.26 | 1.57 | 0.45 |
|  | LA HC | Observed | 0.00 | 1.00 | 5.00 | 9.92 | 9.00 | 71.00 | 288.08 |
|  |  | Raw SIR | 0.00 | 0.40 | 0.53 | 0.64 | 0.73 | 4.09 | 0.62 |
|  | HA HC | Observed | 0.00 | 1.00 | 5.00 | 9.92 | 9.00 | 71.00 | 288.08 |
|  |  | Raw SIR | 0.00 | 0.30 | 0.50 | 0.61 | 0.96 | 1.51 | 0.23 |
| 100 | LA LC | Observed | 0.00 | 0.00 | 0.00 | 0.94 | 1.00 | 7.00 | 1.92 |
|  |  | Raw SIR | 0.00 | 0.00 | 0.00 | 0.55 | 0.98 | 2.85 | 0.49 |
|  | HA LC | Observed | 0.00 | 0.00 | 0.00 | 0.94 | 1.00 | 7.00 | 1.92 |
|  |  | Raw SIR | 0.00 | 0.00 | 0.00 | 0.58 | 1.26 | 1.64 | 0.42 |
|  | LA HC | Observed | 0.00 | 1.00 | 4.00 | 9.97 | 13.25 | 72.00 | 170.13 |
|  |  | Raw SIR | 0.00 | 0.38 | 0.57 | 0.66 | 0.84 | 2.76 | 0.29 |
|  | HA HC | Observed | 0.00 | 1.00 | 4.00 | 9.97 | 13.25 | 72.00 | 170.13 |
|  |  | Raw SIR | 0.00 | 0.28 | 0.75 | 0.67 | 1.08 | 1.47 | 0.20 |
| 225 | LA LC | Observed | 0.00 | 0.00 | 0.00 | 0.96 | 1.00 | 14.00 | 2.47 |
|  |  | Raw SIR | 0.00 | 0.00 | 0.00 | 0.50 | 1.00 | 2.48 | 0.35 |
|  | HA LC | Observed | 0.00 | 0.00 | 0.00 | 0.96 | 1.00 | 14.00 | 2.47 |
|  |  | Raw SIR | 0.00 | 0.00 | 0.00 | 0.52 | 1.06 | 1.93 | 0.36 |
|  | LA HC | Observed | 0.00 | 1.00 | 4.00 | 9.99 | 15.00 | 140.00 | 235.21 |
|  |  | Raw SIR | 0.00 | 0.40 | 0.63 | 0.61 | 0.82 | 2.39 | 0.20 |
|  | HA HC | Observed | 0.00 | 1.00 | 4.00 | 9.99 | 15.00 | 140.00 | 235.21 |
|  |  | Raw SIR | 0.00 | 0.27 | 0.60 | 0.60 | 0.83 | 1.86 | 0.21 |
| 400 | LA LC | Observed | 0.00 | 0.00 | 0.00 | 0.97 | 1.00 | 13.00 | 2.32 |
|  |  | Raw SIR | 0.00 | 0.00 | 0.00 | 0.53 | 1.02 | 3.09 | 0.35 |
|  | HA LC | Observed | 0.00 | 0.00 | 0.00 | 0.97 | 1.00 | 13.00 | 2.32 |
|  |  | Raw SIR | 0.00 | 0.00 | 0.00 | 0.54 | 1.03 | 2.14 | 0.35 |
|  | LA HC | Observed | 0.00 | 1.00 | 5.00 | 9.98 | 13.00 | 130.00 | 219.31 |
|  |  | Raw SIR | 0.00 | 0.46 | 0.65 | 0.64 | 0.84 | 3.00 | 0.19 |
|  | HA HC | Observed | 0.00 | 1.00 | 5.00 | 9.98 | 13.00 | 130.00 | 219.31 |
|  |  | Raw SIR | 0.00 | 0.41 | 0.58 | 0.63 | 0.87 | 2.07 | 0.21 |
| 2500 | LA LC | Observed | 0.00 | 0.00 | 0.00 | 0.95 | 1.00 | 12.00 | 2.19 |
|  |  | Raw SIR | 0.00 | 0.00 | 0.00 | 0.51 | 1.05 | 2.84 | 0.34 |
|  | HA LC | Observed | 0.00 | 0.00 | 0.00 | 0.95 | 1.00 | 12.00 | 2.19 |
|  |  | Raw SIR | 0.00 | 0.00 | 0.00 | 0.52 | 0.99 | 3.30 | 0.41 |
|  | LA HC | Observed | 0.00 | 1.00 | 4.00 | 9.99 | 13.00 | 123.00 | 202.92 |
|  |  | Raw SIR | 0.00 | 0.47 | 0.66 | 0.64 | 0.85 | 2.79 | 0.17 |
|  | HA HC | Observed | 0.00 | 1.00 | 4.00 | 9.99 | 13.00 | 123.00 | 202.92 |
|  |  | Raw SIR | 0.00 | 0.43 | 0.59 | 0.64 | 0.81 | 3.07 | 0.24 |
