# Supplementary material for: Evaluating the impact of a small number of areas on spatial estimation
Source: Int J Health Geogr. 2020 Sep 25;19:39. doi: 10.1186/s12942-020-00233-1 (PMC7519538; doi:10.1186/s12942-020-00233-1)
Supplement: Supplementary file 3 — Additional file 3: Box plots of WAIC for different numbers of areas. [file 12942_2020_233_MOESM3_ESM.docx]

**Additional file 3. Box plots of WAIC for different numbers of areas.**


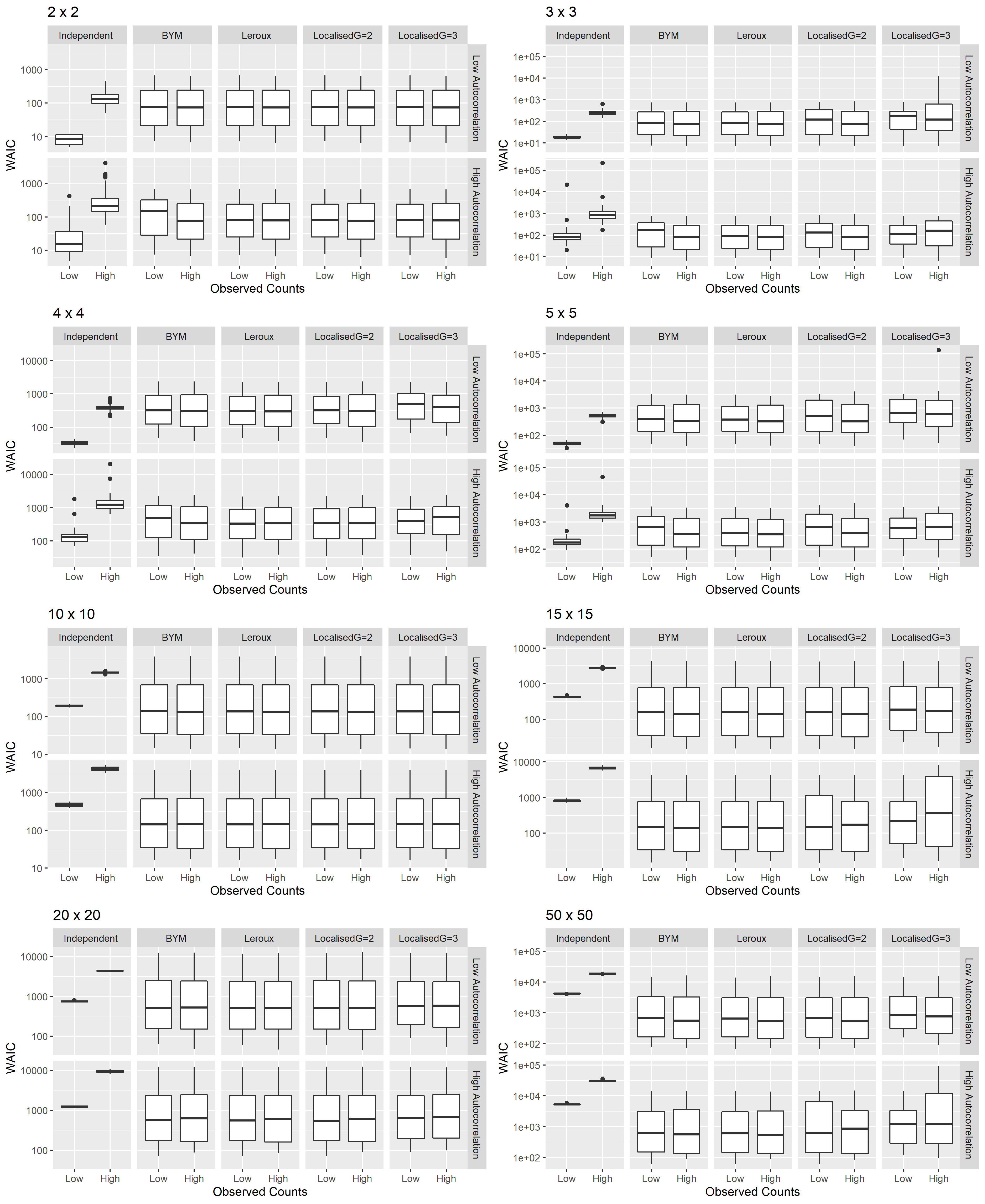


The boxplots show the interquartile range in the box, the range by lines/dots and the mean by the horizontal line in the box with correspond to Table 1 in the manuscript
