# Supplementary material for: Evaluating the impact of a small number of areas on spatial estimation
Source: Int J Health Geogr. 2020 Sep 25;19:39. doi: 10.1186/s12942-020-00233-1 (PMC7519538; doi:10.1186/s12942-020-00233-1)
Supplement: Supplementary file 4 — Additional file 4: The localised structures for localised model with G = 2 and G = 3 in 2002 and 2010; 2011, 2013 and 2015. [file 12942_2020_233_MOESM4_ESM.docx]

**Additional file 4. The localised structures for localised model with G = 2 and G = 3 in 2002 and 2010.**

| 2002 | | | | | | | 2010 | | | | | | |  |
| --- | --- | --- | --- | --- | --- | --- | --- | --- | --- | --- | --- | --- | --- | --- |
| *G* = 2 | | | | *G* = 3 | | | *G* = 2 | | | | *G* = 3 | | |  |
| Districts | DF | SIR | LS | | SIR | LS | | DF | SIR | LS | | SIR | LS | |
| Rappocini | 419 | 2.53 | **2** | | 2.54 | 3 | | 45 | 1.86 | 2 | | 1.87 | 3 | |
| Tamalate | 124 | 0.75 | 1 | | 0.81 | 2 | | 16 | 0.70 | 1 | | 0.73 | 2 | |
| Makassar | 99 | 0.92 | 1 | | 0.95 | 2 | | 9 | 0.71 | 1 | | 0.74 | 2 | |
| Mariso | 79 | 1.14 | 1 | | 1.10 | 2 | | 8 | 0.79 | 1 | | 0.80 | 2 | |
| Mamajang | 93 | 1.12 | 1 | | 1.07 | 2 | | 14 | 1.69 | 2 | | 1.60 | 3 | |
| Ujung Pandang | 39 | 1.00 | 1 | | 1.02 | 2 | | 2 | 0.72 | 1 | | 0.73 | 2 | |
| Tallo | 59 | 0.42 | 1 | | 0.41 | 1 | | 18 | 0.73 | 1 | | 0.76 | 2 | |
| Manggala | 93 | 0.88 | 1 | | 0.90 | 2 | | 6 | 0.70 | 1 | | 0.64 | 2 | |
| Panakkukang | 216 | 1.28 | 1 | | 1.22 | 2 | | 31 | 1.78 | 2 | | 1.74 | 3 | |
| Wajo | 39 | 0.84 | 1 | | 0.94 | 2 | | 1 | 0.71 | 1 | | 0.67 | 2 | |
| Bontoala | 36 | 0.47 | 1 | | 0.44 | 1 | | 8 | 0.82 | 1 | | 0.83 | 2 | |
| Ujung Tanah | 14 | 0.32 | 1 | | 0.36 | 1 | | 3 | 0.71 | 1 | | 0.72 | 2 | |
| Tamalanrea | 62 | 0.61 | 1 | | 0.53 | 1 | | 7 | 0.70 | 1 | | 0.70 | 2 | |
| Biringkanaya | 95 | 0.75 | 1 | | 0.77 | 2 | | 17 | 0.70 | 1 | | 0.73 | 2 | |
| WAIC | 175.08 | | | | 124.54 | | | 79.68 | | 81.8 | | | | |
| DF: Dengue fever; SIR: Standardised incidence ratio; LS: Localised structure | | | | | | | | | | | | | |  |

**Additional file 4. (continued).**

**The localised structures for localised model with G = 2 and G = 3 in 2011, 2013 and 2015.**

| 2011 | | | | | | | 2013 | | | | | | |  |
| --- | --- | --- | --- | --- | --- | --- | --- | --- | --- | --- | --- | --- | --- | --- |
| *G* = 2 | | | | *G* = 3 | | | *G* = 2 | | | | *G* = 3 | | |  |
| Districts | DF | SIR | LS | | SIR | LS | | DF | SIR | LS | | SIR | LS | |
| Rappocini | 16 | 1.15 | 1 | | 1.11 | 2 | | 33 | 0.88 | 1 | | 0.95 | 2 | |
| Tamalate | 7 | 0.97 | 1 | | 1.00 | 2 | | 39 | 0.90 | 1 | | 0.97 | 2 | |
| Makassar | 2 | 0.90 | 1 | | 0.90 | 2 | | 14 | 0.88 | 1 | | 0.95 | 2 | |
| Mariso | 2 | 0.99 | 1 | | 1.02 | 2 | | 11 | 0.90 | 1 | | 0.97 | 2 | |
| Mamajang | 3 | 0.96 | 1 | | 0.98 | 2 | | 17 | 1.13 | 1 | | 1.16 | 2 | |
| Ujung Pandang | 2 | 1.01 | 1 | | 1.03 | 2 | | 9 | 1.20 | 1 | | 1.22 | 2 | |
| Tallo | 10 | 1.00 | 1 | | 1.01 | 2 | | 9 | 0.84 | 1 | | 0.42 | 1 | |
| Manggala | 3 | 0.86 | 1 | | 0.86 | 2 | | 52 | 2.10 | 2 | | 2.08 | 3 | |
| Panakkukang | 16 | 1.19 | 1 | | 1.15 | 2 | | 29 | 0.90 | 1 | | 0.97 | 2 | |
| Wajo | 3 | 1.07 | 1 | | 1.08 | 2 | | 4 | 0.88 | 1 | | 0.92 | 2 | |
| Bontoala | 1 | 0.95 | 1 | | 0.97 | 2 | | 7 | 0.85 | 1 | | 0.90 | 2 | |
| Ujung Tanah | 2 | 0.98 | 1 | | 1.01 | 2 | | 4 | 0.85 | 1 | | 0.77 | 2 | |
| Tamalanrea | 13 | 1.18 | 1 | | 1.14 | 2 | | 13 | 0.87 | 1 | | 0.89 | 2 | |
| Biringkanaya | 5 | 0.79 | 1 | | 0.77 | 2 | | 24 | 0.83 | 1 | | 0.87 | 2 | |
| WAIC | 76.07 | | | | 75.47 | | | 97.24 | | 91.36 | | | | |
| LS: Localised structure | | | | | | | | | | | | | |  |

| 2015 | | | | | | |  |
| --- | --- | --- | --- | --- | --- | --- | --- |
| *G* = 2 | | | | *G* = 3 | | |  |
| Districts | DF | RR | LS | | RR | LS | |
| Rappocini | 17 | 0.99 | 1 | | 0.99 | 2 | |
| Tamalate | 14 | 0.98 | 1 | | 0.98 | 2 | |
| Makassar | 7 | 0.99 | 1 | | 0.99 | 2 | |
| Mariso | 4 | 0.98 | 1 | | 0.98 | 2 | |
| Mamajang | 7 | 0.99 | 1 | | 0.99 | 2 | |
| Ujung Pandang | 2 | 0.99 | 1 | | 0.99 | 2 | |
| Tallo | 17 | 1.00 | 1 | | 1.00 | 2 | |
| Manggala | 9 | 1.00 | 1 | | 0.99 | 2 | |
| Panakkukang | 14 | 0.98 | 1 | | 0.98 | 2 | |
| Wajo | 7 | 0.99 | 1 | | 0.99 | 2 | |
| Bontoala | 2 | 0.97 | 1 | | 0.98 | 2 | |
| Ujung Tanah | 2 | 0.97 | 1 | | 0.98 | 2 | |
| Tamalanrea | 14 | 0.99 | 1 | | 1.00 | 2 | |
| Biringkanaya | 26 | 1.06 | 1 | | 1.06 | 2 | |
| WAIC | 73.29 | | | | 73.39 | | |
