# Supplementary material for: Evaluating the impact of a small number of areas on spatial estimation
Source: Int J Health Geogr. 2020 Sep 25;19:39. doi: 10.1186/s12942-020-00233-1 (PMC7519538; doi:10.1186/s12942-020-00233-1)
Supplement: Supplementary file 5 — Additional file 5: The number of groups forming under every scenario. [file 12942_2020_233_MOESM5_ESM.docx]

Additional file 5. The number of groups forming under every scenario

|  | Localised G =2 | | | | Localised G = 3 | | | |
| --- | --- | --- | --- | --- | --- | --- | --- | --- |
|  | LA LC | HA LC | LA HC | HA HC | LA LC | HA LC | LA HC | HA HC |
| 2 × 2 | 1 | 1 | 2 | 2 | 1 | 1 | 2 | 2 |
| 3 × 3 | 1 | 1 | 2 | 2 | 1 | 1 | 2 | 2 |
| 4 × 4 | 1 | 1 | 2 | 2 | 1 | 1 | 2 | 2 |
| 5 × 5 | 1 | 1 | 2 | 2 | 1 | 1 | 2 | 1 |
| 10 × 10 | 1 | 1 | 1 | 1 | 1 | 1 | 1 | 1 |
| 15 × 15 | 1 | 1 | 1 | 2 | 1 | 1 | 2 | 1 |
| 20 × 20 | 1 | 1 | 1 | 2 | 1 | 1 | 2 | 1 |
| 50 × 50 | 1 | 1 | 1 | 1 | 1 | 1 | 1 | 1 |
